# First application of dynamic oxygen-17 magnetic resonance imaging at 7 Tesla in a patient with early subacute stroke

**DOI:** 10.3389/fnins.2023.1186558

**Published:** 2023-06-15

**Authors:** Louise Ebersberger, Fabian J. Kratzer, Arne Potreck, Sebastian C. Niesporek, Myriam Keymling, Armin M. Nagel, Martin Bendszus, Wolfgang Wick, Mark E. Ladd, Heinz-Peter Schlemmer, Angelika Hoffmann, Tanja Platt, Daniel Paech

**Affiliations:** ^1^Division of Radiology, German Cancer Research Center (DKFZ), Heidelberg, Germany; ^2^Faculty of Medicine, University of Heidelberg, Heidelberg, Germany; ^3^Department of Pediatrics, Bern University Hospital, Bern, Switzerland; ^4^Division of Medical Physics in Radiology, German Cancer Research Center (DKFZ), Heidelberg, Germany; ^5^Department of Neuroradiology, Heidelberg University Hospital, Heidelberg, Germany; ^6^Institute of Radiology, Friedrich-Alexander University Erlangen-Nürnberg (FAU), Erlangen University Hospital, Erlangen, Germany; ^7^Department of Neurology, Heidelberg University Hospital, Heidelberg, Germany; ^8^Faculty of Physics and Astronomy, University of Heidelberg, Heidelberg, Germany; ^9^Department of Neuroradiology, Bern University Hospital, Bern, Switzerland; ^10^Department of Neuroradiology, Bonn University Hospital, Bonn, Germany

**Keywords:** oxygen-17 MRI, stroke, metabolic imaging, ultra-high field, 7 Tesla, oxygen metabolism

## Abstract

Dynamic oxygen-17 (^17^O) magnetic resonance imaging (MRI) is an imaging method that enables a direct and non-invasive assessment of cerebral oxygen metabolism and thus potentially the distinction between viable and non-viable tissue employing a three-phase inhalation experiment. The purpose of this investigation was the first application of dynamic ^17^O MRI at 7 Tesla (T) in a patient with stroke. In this proof-of-concept experiment, dynamic ^17^O MRI was applied during ^17^O inhalation in a patient with early subacute stroke. The analysis of the relative ^17^O water (H_2_^17^O) signal for the affected stroke region compared to the healthy contralateral side revealed no significant difference. However, the technical feasibility of ^17^O MRI has been demonstrated paving the way for future investigations in neurovascular diseases.

## 1. Introduction

According to the World Health Organization, stroke is a common cause of disability and currently the second leading cause of death world-wide (The top 10 causes of death, 2020). This disease is not only deadly, but is a common cause of disability as it often entails permanent neurological deficits with enormous impact on the patient’s quality of life (Hong, 2011). Despite the improvement of patient outcome with mechanical recanalization, patients still benefit from the therapy to a variable degree. Neuroimaging before, but also after recanalization may support outcome prediction by, e.g., distinguishing viable tissue from unviable tissue.

Oxygen extraction fraction magnetic resonance imaging (MRI) is a promising technique for identification of at-risk tissue in stroke (Fan et al., 2020). However, this imaging technique only provides indirect information on tissue oxygenation, unlike oxygen-15 (^15^O) positron emission tomography (PET). Even though ^15^O PET is considered the gold standard for oxygen imaging, having detected ischemic penumbra *in vivo* for the first time (Zaro-Weber et al., 2019), clinical routine imaging with this method is challenging due to its complexity and long measurement duration as well as the use of the radioactive ^15^O isotope with very short half-life ( ~ 123 s) (Fox and Raichle, 1986; Delapaz and Gupte, 2011).

Oxygen-17 (^17^O) imaging makes use of the MR properties of the non-radioactive oxygen isotope ^17^O, which can be applied during the MR experiment either by administration of ^17^O-labeled water or by inhalation of ^17^O gas (^17^O_2_). The first *in vivo* ^17^O experiments were performed three decades ago with indirect (Hopkins and Barr, 1987) and direct (Arai et al., 1990, 1991; Pekar et al., 1991) detection of physiological properties. The indirect ^17^O MR approaches are based on the detection of changes in T2- or T1 ρ -weighted proton NMR signals caused by ^17^O-^1^H scalar coupling and chemical exchange (Zhu et al., 2005), while the direct ^17^O MR approach measures ^17^O water (H_2_^17^O) itself. In preclinical studies, ^17^O MRI has been used to study various species including mouse, rat, cat and swine at field strengths between 3 Tesla (T) and 16.4 T (Zhu and Chen, 2017). A ^17^O MRI approach for studying the cerebral oxygen metabolism was developed for rats about two decades ago (Zhu et al., 2002). Since then, several pathologies have been studied in mice using ^17^O MRI, such as amyloidosis (Baligand et al., 2021) and Huntington’s disease (Lou et al., 2016). The safe and feasible application of indirect (Harada et al., 2022) and direct ^17^O MRI (Fiat et al., 1993, 2004), and ^17^O MR spectroscopy (Zhu et al., 2005) to humans has been demonstrated. The metabolic model presented by Atkinson and Thulborn (2010) paved the way for the three-phase ^17^O_2_ inhalation experiments used in dynamic ^17^O MRI. This methodical setup measures the metabolized H_2_^17^O that accumulates during cellular respiration, while the ^17^O_2_ gas does not contribute to the measured signal.

Hence, dynamic ^17^O MRI enables the direct and non-invasive assessment of cerebral oxygen metabolism (Atkinson and Thulborn, 2010), and could thus directly measure oxygen consumption of hypoperfused ischemic tissue. In clinical research, dynamic ^17^O MRI has primarily been used for brain tumor imaging, confirming the Warburg theorem of lower oxygen metabolism in cancer (Hoffmann et al., 2014; Paech et al., 2020). Dynamic ^17^O MRI is a tool that can reflect the aerobic oxygen metabolism, hence the potential for clinical stroke imaging has been hypothesized on several occasions. As early as 2011, the application of dynamic ^17^O MRI to cerebral ischemia in humans has been proposed by Delapaz and Gupte (2011). Two years later a preclinical study by Zhu et al. (2013) demonstrated the successful application of ^17^O MRI for stroke imaging in a mouse model and suggested the possibility of extending the method to stroke patients. In 2020, Rapalino highlighted the prospect of this modality for investigating cerebral ischemia (Rapalino, 2020). Although ^17^O MRI has been postulated to be a promising research tool for stroke imaging, cerebral ischemia has not yet been studied in humans with this method.

The scope of this study was to evaluate the feasibility of dynamic ^17^O MRI for application in stroke. In this proof-of-concept study, we included one patient with early subacute stroke caused by vasculitis, employing dynamic ^17^O MRI at 7 T.

## 2. Patient and methods

### 2.1. Patient

**Figure 1:**
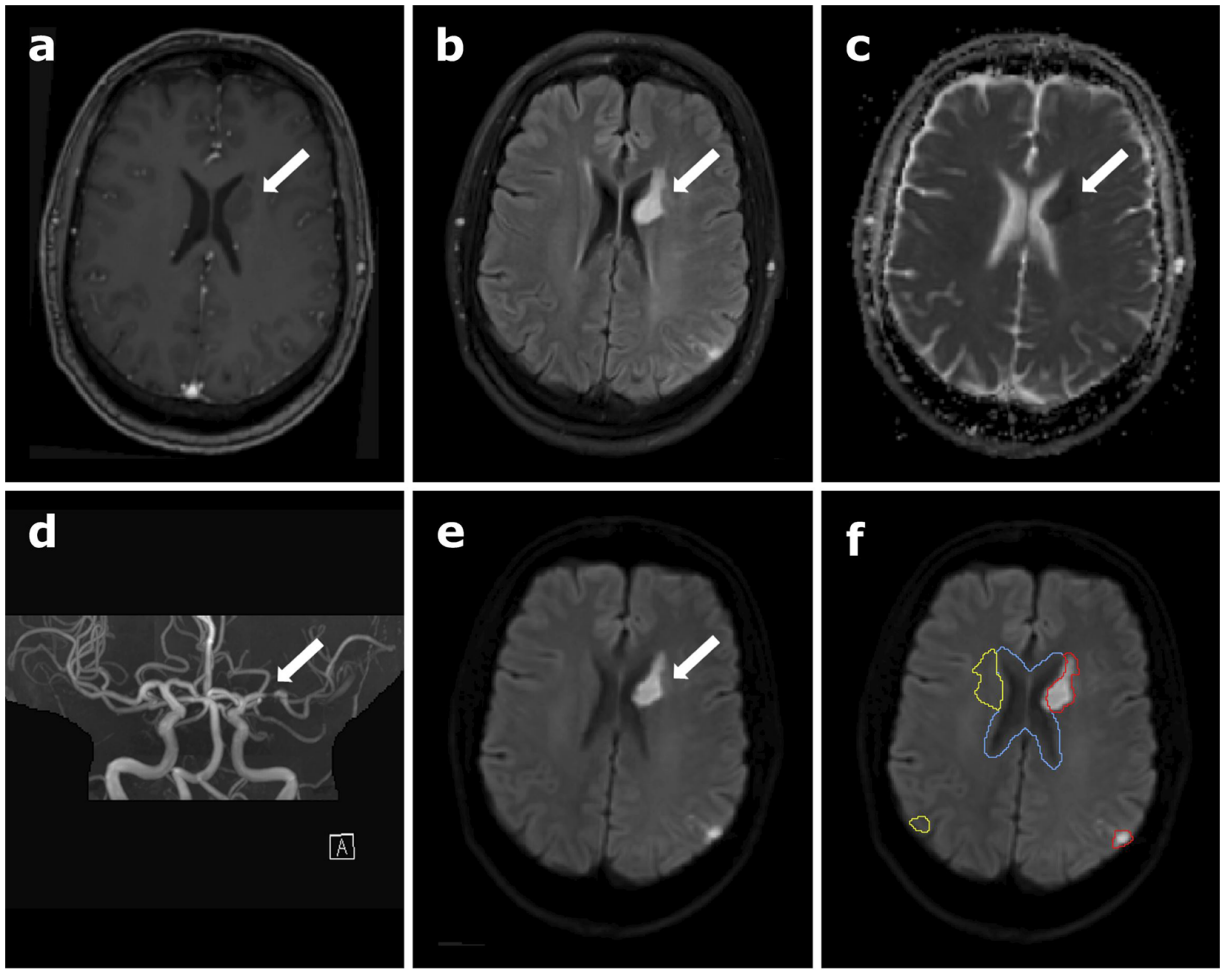
Clinical data of the patient depicting the stroke region, **(A)** contrast-enhanced T1-weighted magnetization prepared rapid gradient echo (MPRAGE) data, **(B)** T2-weighted fluid-attenuated inversion recovery (T2 FLAIR), **(C)** apparent diffusion coefficient (ADC) map calculated from diffusion-weighted imaging (DWI), **(D)** time-of-flight angiography (TOF), **(E)** B1000 DWI, and **(F)** B1000 DWI overlaid with the different regions of interest (ROI) used for data analysis in oxygen-17 (^17^O) MR data: the red contour outlines the stroke region, and cerebral spinal fluid (CSF) is delineated in blue. The yellow contour shows the mirrored stroke ROI, comprising healthy brain tissue. The white arrows indicate the location of the stroke.

One patient (male, age 55) was included for this proof-of-concept study. The patient had a known history of vasculitis and had been diagnosed with acute stroke in the left middle cerebral artery territory, affecting the basal ganglia and a small region of the parietal-occipital cortex, see Figure 1. Computer tomography and standard clinical MR images at 3 T for anatomical and diffusion imaging had been performed immediately upon admission to the University Hospital Heidelberg, Germany. The dynamic ^17^O MRI at 7 T was performed in the early subacute state, 5 days later. At the time of the oxygen imaging the patient was not experiencing any severe neurological deficits (National Institutes of Health Stroke Scale (NIHSS): 1). Prior to the examination, written informed consent was obtained in accordance with the institutional guidelines and the study was approved by the ethics committee of the Medical Faculty Heidelberg, Germany (S-154/2014).

### 2.2. Materials and methods

#### 2.2.1. MRI protocol and dynamic ^17^O experiment

The clinical magnetic resonance (MR) data were acquired at 3 T employing a whole-body system (Prisma; Siemens Healthcare, Erlangen, Germany) and included the standard protocol for stroke imaging at the local department for neuroradiology, including T2-weighted fluid-attenuated inversion recovery (T2-FLAIR), T2 turbo spin echo (TSE), susceptibility-weighted imaging (SWI), time-of-flight (TOF), diffusion-weighted imaging (DWI), and T1-weighted magnetization prepared rapid gradient echo (MPRAGE) pre and post contrast administration.

The oxygen data were obtained on a 7 T whole-body MR system (Magnetom 7 T; Siemens Healthcare, Erlangen, Germany) using a home-built ^17^O birdcage head coil with an additional proton (^1^H) channel (Hoffmann et al., 2014; Niesporek et al., 2018). At 7 T, the total duration of the patient measurement amounted to approximately 40 min, including 30 min (shortened patient protocol) (Paech et al., 2020) of dynamic ^17^O imaging (TE/TR = 0.56 ms/20 ms, flip angle: 60°, t_pulse_ = 1 ms, readout duration = 5.5 ms, nominal resolution: (7.5 mm)^3^, number of projections: 90000) applying a density-adapted radial pulse sequence (Nagel et al., 2009) and 10 min acquisition of a gradient echo (GRE) image (TE/TR = 3.25 ms/7.5 ms, flip angle: 10°, matrix size: 256x256x176, nominal resolution: (1 mm)^3^) for registration. To estimate the effective resolution of the oxygen images, the point spread function (PSF) was simulated considering the readout trajectory, T_1_ and T_2_* relaxation [T_1_ = 5 ms, WM: T_2_* = 2.8 ms, GM: T_2_* = 2.5 ms, CSF: T_2_* = 5 ms (Niesporek et al., 2018)] and the reconstruction filter (Hamming). T_2_* bias for the different tissues was estimated by calculating the signal at the echo time TE = 0.56 ms: $e^{-\frac{TE}{T_{2}^{*}}}$ .

The ^17^O imaging for the healthy volunteers was 40 min (number of projections: 120000, see Supplementary material). A sliding window reconstruction was applied to the ^17^O data sets (per image: 3000 projections, acquisitions time: 1 min; patient: 30 images, volunteers: 40 images).

The ^17^O measurement is a dynamic experiment with three inhalation phases: during the first phase, the patient breathes regular room air through an MR-compatible breathing mask (baseline phase, 5 min). Subsequently, the inhalation system is switched to a reservoir containing approximately 4 L of 70% ^17^O_2_-enriched oxygen gas, starting the second phase (inhalation phase, approximately 6 min). Then, a second switch back to room air initiates the last phase, which lasts until 30 min of continuous MR data are acquired. The details concerning the experimental set up have been described in previous studies (Niesporek et al., 2018; Paech et al., 2020).

#### 2.2.2. Image registration and segmentation

For the 7 T data, the GRE images were registered to the ^17^O images automatically using the FLIRT algorithm of FSL (FMRIB Software Library) (Jenkinson and Smith, 2001; Jenkinson et al., 2002). The co-registration of the clinical 3 T images to the 7 T GRE images was conducted by manual pre-registration followed by an automatic registration, again using the FLIRT algorithm. Masks for cerebral gray matter (GM), cerebral white matter (WM) and cerebral spinal fluid (CSF) were obtained by applying the FAST segmentation tool for an automatic segmentation to the MPRAGE data set (Zhang et al., 2001). Further regions-of-interest (ROIs) included the stroke area (striatal and cortical stroke together), the mirrored control area and the ventricles, which were segmented manually in the Medical Imaging Interaction Toolkit (MITK) (Wolf et al., 2005; Nolden et al., 2013) according to the B1000 diffusion images, see Figure 1F. These segmentations were performed by two readers in consensus, reader 1 (L.E.) with 2 years and reader 2 (D.P.) with 10 years of experience in neuroimaging and were applied for further investigations. To check for inter-reading variability, a second reading has been performed by reader 1, blinded to the original segmentation (see Supplementary material).

#### 2.2.3. Data analysis

A binary mask based partial volume correction (PVC) was performed on the oxygen data set, as proposed by Niesporek et al. (2015, 2018), which applies the geometric transfer matrix (GTM) PVC method. This algorithm takes into account anatomical information using the tissue masks for GM, WM, and CSF and for the stroke area as well as the simulated tissue-specific PSF. This approach provides a region-specific PV-corrected signal value within each mask and for each time point.

Additionally, the relative H_2_^17^O signal evolution was investigated without PVC within different ROIs: ventricles, stroke area, mirrored control and the remaining healthy brain tissue (whole brain minus ROIs of stroke area, mirrored control and ventricles). Since the stroke area lies in proximity to the ventricles, the influence of partial volume effects on the stroke area was minimized for this analysis without PVC by dilating the ventricle ROI twice using the segmentation utilities tool in MITK and subtracting the overlap from the stroke and mirrored control ROIs, resulting in a smaller stroke ROI.

To obtain the relative curves, all data points were normalized to the mean of the baseline (5 min). The relative H_2_^17^O signal evolutions of the patient were compared to those of three healthy volunteers (all male, ages 28, 37 and 65), each measured twice with ^17^O MRI at 7 T in the past (Niesporek et al., 2018). The data analysis and the testing procedure for the healthy volunteers can be found in the Supplementary material. For all measurements, five data points around the second switch from ^17^O_2_ inhalation back to room air, thus the point of longest ^17^O_2_ inhalation, were averaged and compared. Additionally, the standard deviation was calculated for the first five data points of the baseline.

Furthermore, the relative ^17^O signal increase is obtained by subtracting a baseline image during breathing of room air from a ^17^O image at the end of ^17^O_2_ inhalation (maximum signal); then dividing the result by the baseline image (Paech et al., 2020). Data of the two images were acquired in 3 min each. For smoothing of these relative ^17^O images and for obtaining a refined image resolution, a Hamming filter was applied, as well as eightfold zero-filling.

## 3. Results

### 3.1. Effective resolution of oxygen images and T_2_* influences

The simulated full width half maximum (FWHM) of the PSF is approximately 2.3 voxels, which results in an effective resolution of the oxygen images of *circa* ((17–17.5)mm)^3^ for WM and GM. For CSF, the FWHM is approximately 2.2 voxels and the effective resolution (16 mm)^3^. Due to the T_2_* decay the signal at TE for the different tissues was estimated to be: CSF: 100%, WM: 92%, GM: 89%.

### 3.2. Data analysis

**Figure 2:**
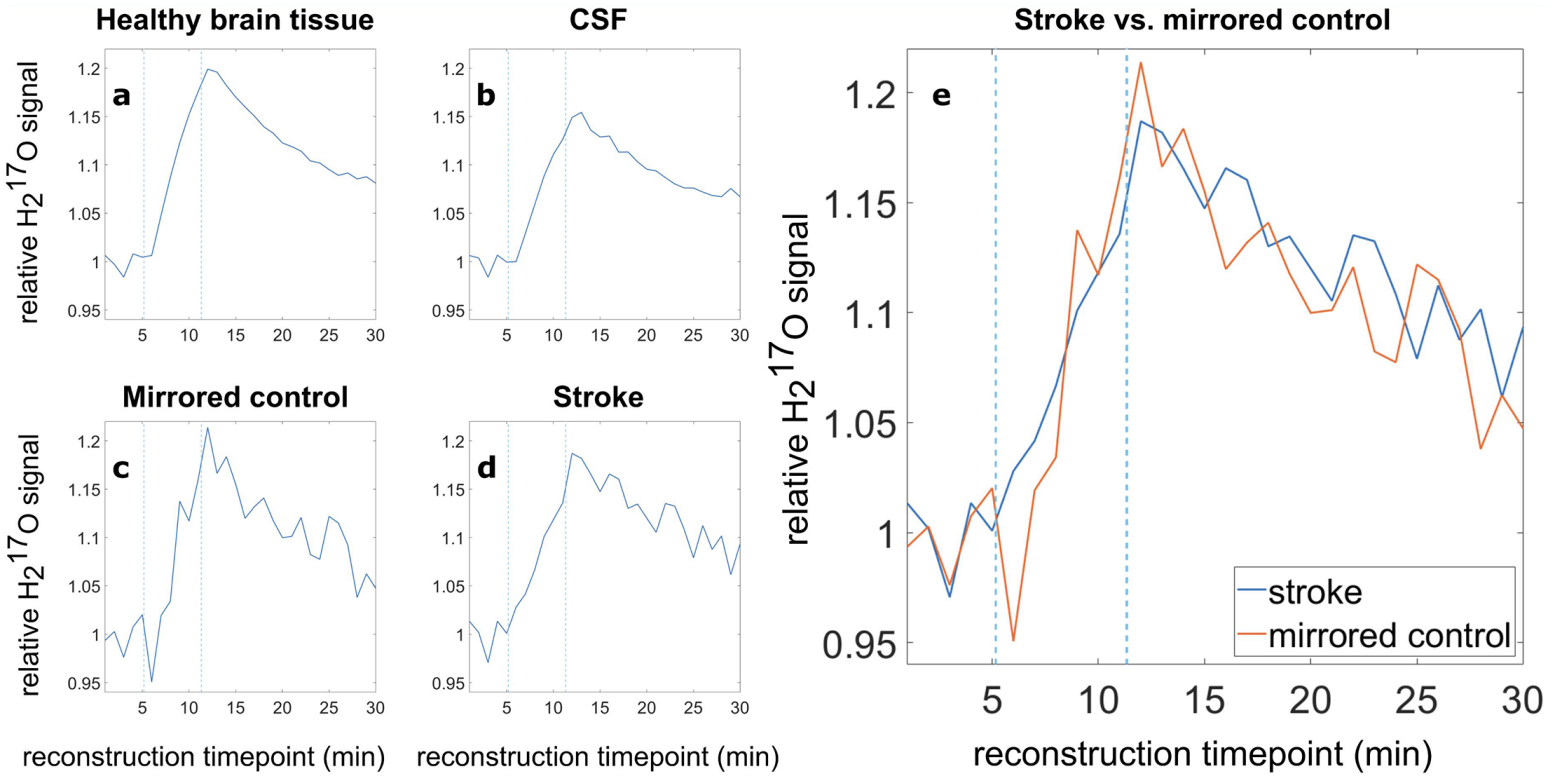
Relative ^17^O water (H_2_^17^O) signal evolution in various tissue types, **(A)** healthy brain tissue, **(B)** CSF, **(C)** the mirrored control, **(D)** the stroke region, and **(E)** direct comparison of the relative H_2_^17^O signal in the stroke ROI (blue) and the mirrored contralateral healthy ROI (orange). Blue dashed lines indicate the switching times from room air to ^17^O-enriched gas and back to room air.

The relative PV-corrected time evolution in the stroke ROI is depicted in Supplementary Figure S2 in the Supplementary material in comparison to the time evaluation without PVC. The PV-corrected data shows a high noise level. Relative H_2_^17^O signal evolutions without PVC for healthy brain tissue, CSF, stroke ROI and mirrored control are depicted in Figure 2. The evaluation of the relative H_2_^17^O signal evolution revealed no significant difference between the stroke area and the mirrored control area.

The relative signal after the inhalation phase was analyzed by averaging five data points (5 min) around the time of the second switch. Here, the healthy brain tissue, encompassing the complete brain tissue minus the ventricles, stroke and mirrored control ROI, showed a relative mean signal (5 data points around the second switching time, evenly spaced 60 s apart, corresponding to 5 min in total) of 1.181 (Figure 2A). The evaluation for CSF resulted in a relative mean H_2_^17^O signal of 1.135 (Figure 2B). Due to the smaller ROI size, the stroke and the mirrored control region, Figures 2C,D show larger signal fluctuations compared to the relative curves of healthy brain tissue and CSF (Figures 2A,B). The evaluation of the relative H_2_^17^O signal in the stroke ROI yielded a relative mean signal (5 data points around the second switching time) of 1.158, while the mirrored control exhibited a relative mean H_2_^17^O signal of 1.168.

For better comparability, the relative oxygen signal evolution for stroke (Figure 2D), and its mirrored control (Figure 2C), were overlaid in Figure 2E. Averaging five data points around the time of the second switch, the signal increase in the stroke area is about 0.9% less than in the mirrored control. The analysis of six data sets obtained from three healthy volunteers showed differences between −1.9 and + 0.3% between the left ROI (equivalent to stroke) and right ROI (equivalent to mirrored control). Furthermore, the standard deviation in the baseline (relative to the mean) in the healthy subjects is 2.2% (left) and 2.3% (right), respectively. The detailed analysis is shown in Supplementary Tables S1, S2 and Supplementary Figure S3 in the Supplementary material.

**Figure 3:**
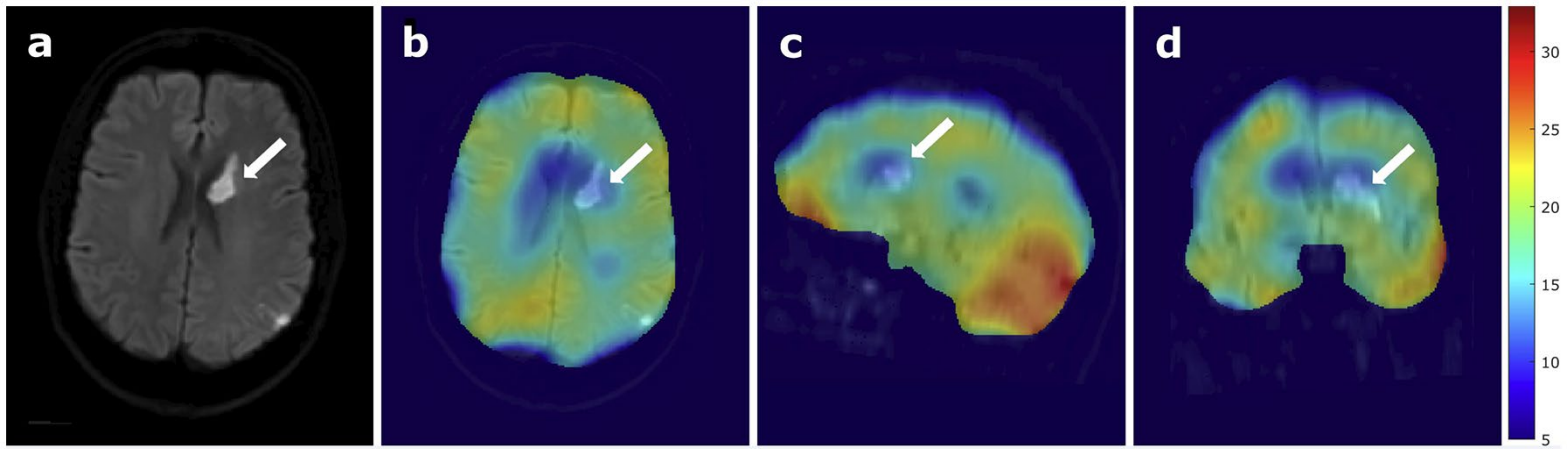
^17^O MRI at 7 Tesla in a patient with stroke, **(A)** B1000 DWI of the patient only and **(B)** overlaid with relative ^17^O images, showing the stroke region in an axial view, **(C)** sagittal view, and **(D)** coronal view. The white arrows indicate the location of the stroke.

In the relative ^17^O images, the lateral ventricles containing CSF show a low ^17^O signal increase, indicating a region with low metabolic activity. The stroke area exhibits a similarly low ^17^O signal increase, indicated by a white arrow in Figure 3. However, also the mirrored control area shows a low ^17^O signal increase. The outer cortex regions show a rather homogenous relative signal distribution. The cerebellum exhibits a high ^17^O signal increase, indicating high metabolic activity.

## 4. Discussion

### 4.1. Data interpretation

In this proof-of-concept study, dynamic ^17^O MRI was applied for the first time during ^17^O inhalation in a patient with early subacute stroke.

Firstly, the PV-corrected relative H_2_^17^O signal evolution of the stroke area, corresponding to a very small ROI, showed high signal fluctuations. The noise level impeded the reliable interpretation of changes in the oxygen signal evolution, as they cannot be confidently attributed to regionally altered metabolic activity. Further investigations with PVC might be possible in patients with larger stroke areas. However, the patient’s condition will likely be worse and might not allow for dynamic ^17^O MRI.

Secondly, the evaluation of the relative H_2_^17^O signal evolution in the patient revealed no significant difference between the stroke area and the mirrored control area. The deviation between stroke and mirrored control in the patient was in the same order of magnitude as in the healthy volunteers, and in the same order of magnitude as the calculated standard deviation in the volunteer data sets during the baseline (breathing room air).

The relative ^17^O images showed visual differences between the cortex regions, the cerebellum and the CSF, as shown in the literature. The CSF exhibited a low ^17^O signal, since it does not contain cells, but instead drains the metabolized H_2_^17^O water from the surrounding brain tissue (Paech et al., 2020). The relative images showed lower signal increase in the stroke area but also in the mirrored control areas, suggesting a lower metabolic activity within this region, yet this might be due to spillover effects from the adjacent ventricles. This spill over is a result of the FWHM of the PSF of *circa* 2.2–2.3 voxels. Additionally, if signal contributions from CSF and brain tissues are measured in the same voxel, the quantification can be slightly biased towards the CSF value due to its longer T_2_* of 5 ms (GM/WM: 89/92% of CSF signal).

The influence of the segmentation variability of the stroke area on these results has been considered. However, the high dice coefficient of 86.3% for the two readings performed for the stroke ROI, together with the relatively low resolution of the oxygen images of [(17–17.5) mm]^3^, lead to the conclusion that slight changes of the segmentations on the high-resolution proton images do not markedly affect the evaluation of the ^17^O data.

These findings demonstrated the general feasibility of employing dynamic ^17^O MRI in a patient with stroke, but did not show a significant quantitative difference between the stroke region and the mirrored control in this particular patient.

This motivates future investigations in patients with larger strokes that are not located in vicinity to the ventricles. A ^15^O PET study in patients with acute stroke showed that the investigation of a stroke in the basal ganglia near the CSF afforded less prominent results in comparison to larger strokes located in the cortex (Zaro-Weber et al., 2019). Thus, dynamic ^17^O MRI remains a promising imaging technique for detection of changes in oxygen metabolism that are possibly associated with neuronal impairment or death.

The direct comparison to other metabolic imaging techniques, first and foremost ^15^O PET, would be especially interesting as well as the investigation of acute stroke with dynamic ^17^O MRI. In clinical routine MRI is more feasible than PET and less complex for stroke imaging (Zaro-Weber et al., 2019). However, the overestimation of both penumbra and ischemic core poses a problem when using mismatch imaging (Delapaz and Gupte, 2011), so that further development of novel imaging methods remains crucial for the improvement of stroke diagnostics.

In this study, we were able to conduct the first 7 T dynamic ^17^O experiment in a study participant with early subacute stroke. The results of this study did not show a significant difference between the stroke region and the mirrored healthy contralateral side, motivating the investigation of patients with a larger stroke area, preferably with location in the cortex. Technical advances including even higher magnetic field strengths and improvement of hardware equipment could boost the resolution of dynamic ^17^O MRI and might pave the way for clinical application in stroke in the future (Platt et al., 2021).

### 4.2. Limitations

This study demonstrated the feasibility of employing ^17^O MRI in a patient with early subacute stroke. For better assessment, further examinations in patients with stroke would be beneficial. The investigated cerebral ischemia in the basal ganglia was relatively small and in proximity to the lateral ventricles. Since the CSF itself does not exhibit metabolic activity, this is an area of low metabolic turnover. Due to partial volume effects, the adjacent CSF might bias the metabolic signal within the stroke ROI. However, prior extraction of the CSF region with a security margin and the use of a mirrored control region on the contralateral side reduced this bias. The measurement of a larger cerebral infarction would probably yield more distinct results, but these patients would be rather instable for this complex examination. Furthermore, the measurement at 7 T was conducted in the early subacute stroke time frame. The investigation of (hyper-)acute stroke within less than 48 h after symptom onset might provide more information concerning the viability of the hypo- or hyperperfused tissue, but would be even more challenging in the clinical setting. However, continuous monitoring of the patient in the stroke unit is recommended for the first 48 h, which impedes research studies in this time frame.

As for the comparison of the clinical data to the three data sets of healthy participants, the demographics are quite similar which allows for fair comparability between the data sets. All investigated subjects are male and the patient’s age (55 years) lies between the age of volunteer 1 (age 65) and 2 (age 37).

## Data availability statement

The raw data supporting the conclusions of this article are available only upon scientific request because patient data are included. The corresponding author (DP) may be contacted to request the data.

## Ethics statement

The studies involving human participants were reviewed and approved by IRB committee, University of Heidelberg, Germany. The patients/participants provided their written informed consent to participate in this study.

## Author contributions

LE, AP, AH, TP, and DP: study conceptualization. LE, FK, SN, AN, TP, and DP: technical methodology. LE, AP, MK, AH, and TP: patient examinations. LE, FK, AN, TP, and DP: data processing. LE, AN, TP, and DP: statistical analysis. LE: writing first draft. LE, FK, AP, SN, MK, AN, MB, WW, ML, H-PS, AH, TP, and DP: reviewing and editing first draft. AN, MB, WW, ML, H-PS, AH, TP, and DP: scientific supervision. All authors contributed to the article and approved the submitted version.

## Conflict of interest

The authors declare that the research was conducted in the absence of any commercial or financial relationships that could be construed as a potential conflict of interest.

## Publisher’s note

All claims expressed in this article are solely those of the authors and do not necessarily represent those of their affiliated organizations, or those of the publisher, the editors and the reviewers. Any product that may be evaluated in this article, or claim that may be made by its manufacturer, is not guaranteed or endorsed by the publisher.
