# Supplementary material for: First application of dynamic oxygen-17 magnetic resonance imaging at 7 Tesla in a patient with early subacute stroke
Source: Front Neurosci. 2023 Jun 15;17:1186558. doi: 10.3389/fnins.2023.1186558 (PMC10317041; doi:10.3389/fnins.2023.1186558)
Supplement: Supplementary file 1 [file Data_Sheet_1.docx]

Supplementary Material

First application of dynamic oxygen-17 (^17^O) magnetic resonance imaging at 7 Tesla in a patient with early subacute stroke

Louise Ebersberger, Fabian J. Kratzer, Arne Potreck, Sebastian C. Niesporek, Myriam Keymling, Armin M. Nagel, Martin Bendszus, Wolfgang Wick, Mark E. Ladd, Heinz-Peter Schlemmer, Angelika Hoffmann, Tanja Platt† & Daniel Paech†*

*** Correspondence:**PD Dr. Daniel Paech, MD, PhD
d.paech@dkfz.de

**† These authors have contributed equally to this work and share last authorship**

# Supplementary Data

**Inter-reading variability in segmentation of the stroke area**

To check for inter-reading variability in the segmentation of the stroke area (striatal and cortical stroke together), two readings have been performed: One performed by two readers in consensus, reader 1 (L.E.) with two years and reader 2 (D.P.) with 10 years of experience in neuroimaging and one by reader 1, blinded to the original segmentation. An exemplary slice of the B1000 data set with the two readings of the stroke area overlapped is depicted in Figure S1. The calculated dice coefficient of the two readings is 86.3%.‬‬‬‬‬‬‬

**Analysis of relative H_2_^17^O signal evolution in healthy volunteers**

In order to enable a better interpretation of the patient data, a total of six data sets from three healthy volunteers, each measured twice [14], have been analyzed. MPRAGE images of these volunteers were registered automatically onto the GRE images, which were acquired in the same position as the ^17^O images, using the FLIRT algorithm of FSL

As previously described in the manuscript, five data points (5 minutes) from the relative H_2_^17^O signal evolutions around the second switch from ^17^O_2_ inhalation back to room air, in literature referred to as TB [7], were averaged and named “max_left_” and “max_right_”, respectively. Additionally, the difference between the two ROIs was calculated in percent (Δmax_LR_ = (max_left_ - max_right_) / max_right_). The results for the healthy volunteers and the patient are shown in **Table S1**.

The analysis of the patient data showed a 0.9% lower average in the stroke area in comparison to the mirrored control. However, the analysis of the volunteer data showed differences between +0.3 and -1.9%, leading to the conclusion that the difference in the stroke data is not significant.

Furthermore, the standard deviation (std) and the coefficient of variation in percent (%CV = std/mean∙100) were calculated for the first five data points of the baseline, giving an estimation for the overall noise level, as the baseline should be constant in theory. The results are shown in **Table S2**.

For the stroke patient, the coefficient of variation of first five data points of the baseline resulted in 1.75% for the stroke ROI and 1.64% for the mirrored control ROI. For the six healthy volunteer measurements, the mean coefficient of variation was 2.16% for the left ROI and 2.27% for the right ROI. This leads to the conclusion that for the stroke patient, the difference in average (Δmax_LR_) of 0.9% between stroke and mirrored control ROI is not significant.

The relative H_2_^17^O signal evolutions for all six data sets of the healthy volunteers and the data of the stroke patient are shown together in **Figure S3** for comparison. The volunteer measurements lasted 40 minutes. For the patient the total measurement time and the inhalation time were shortened, since the ^17^O inhalation experiment is quite demanding for a person with early subacute stroke. The shorter ^17^O inhalation time for the patient leads to less accumulation of metabolic production of H_2_^17^O causing lower averages for the patient compared to the healthy volunteers.

# Supplementary Figures and Tables

## Supplementary Tables

**Table S1. Five data points (5 minutes) around the second switching time were averaged and compared for healthy volunteers (age: volunteer 1: 65, volunteer 2: 38, volunteer 3: 27) and a patient (age: 55).**

| subject | max_left_  "stroke" (left) | max_right_  "mirrored" (right) | Δmax_LR_, % |
| --- | --- | --- | --- |
| volunteer 1.1 | 1.3574 | 1.3837 | -1.90 |
| volunteer 1.2 | 1.3312 | 1.3332 | -0.15 |
| volunteer 2.1 | 1.3952 | 1.4047 | -0.68 |
| volunteer 2.2 | 1.3371 | 1.3333 | 0.29 |
| volunteer 3.1 | 1.3031 | 1.3041 | -0.08 |
| volunteer 3.2 | 1.3218 | 1.3212 | 0.05 |
|  |  |  |  |
| patient | 1.1577 | 1.1684 | -0.92 |

**Table S2. Standard deviation (std) and coefficient of variation (%CV) of first five data points (5 minutes) of the baseline for healthy volunteers and patient.**

| subject | std_baseline_ "stroke" (left) | %CV_baseline_ (left) in % | std_baseline_ "mirrored" (right) | %CV_baseline_ (right) in % |
| --- | --- | --- | --- | --- |
| volunteer 1.1 | 0.0195 | 1.95 | 0.025 | 2.5 |
| volunteer 1.2 | 0.0352 | 3.52 | 0.0288 | 2.88 |
| volunteer 2.1 | 0.0301 | 3.01 | 0.0135 | 1.35 |
| volunteer 2.2 | 0.0263 | 2.63 | 0.0358 | 3.58 |
| volunteer 3.1 | 0.0065 | 0.65 | 0.0152 | 1.52 |
| volunteer 3.2 | 0.0117 | 1.17 | 0.0181 | 1.81 |
| mean |  | 2.16 |  | 2.27 |
|  |  |  |  |  |
| patient | 0.0175 | 1.75 | 0.0164 | 1.64 |

## Supplementary Figures


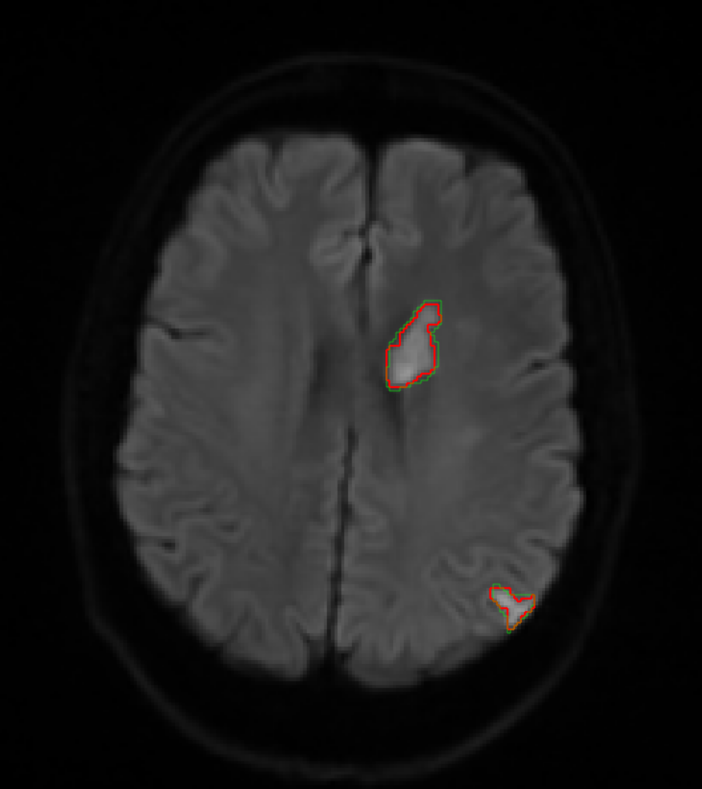


**Figure S1: Exemplary slice of the B1000 DWI of the patient data overlaid with the two independent readings of the stroke area.** Reading 1 is delineated in red, reading 2 is delineated in green.

**
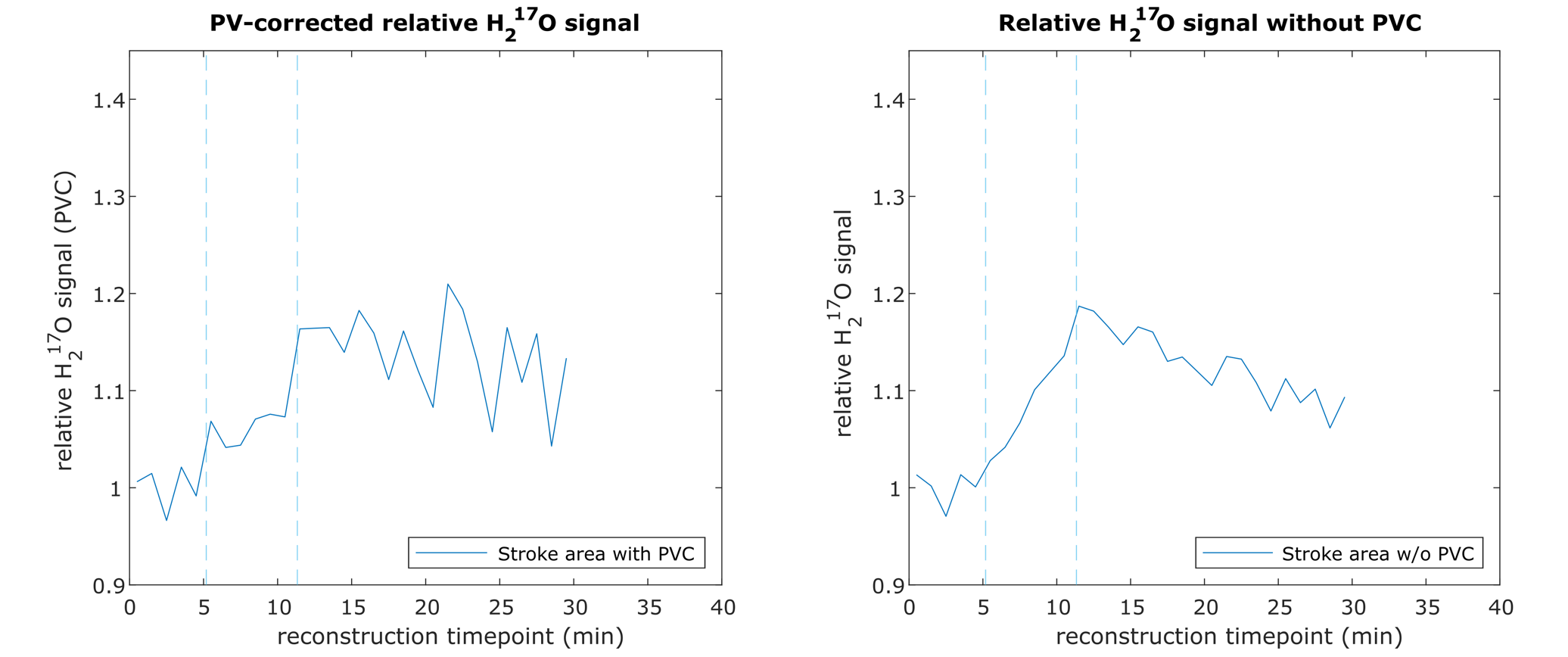
**

**Figure S2:** **PV-corrected time evolution of the relative H_2_^17^O signal in the stroke ROI (left) vs. the time evolution of the relative H_2_^17^O in the stroke ROI without PVC (right).** The signal entries for the PV-corrected time evolution on the left show high signal fluctuations.

# Figure S3: Relative H_2_^17^O signal evolutions of the stroke/left vs. mirrored control/right ROI for the volunteers 1/2/3 and the patient.
